# Supplementary material for: Prenatal Breastfeeding Counseling Intervention in Women with Pre-Gestational Diabetes Mellitus—A Randomized Controlled Trial
Source: Healthcare (Basel). 2024 Feb 4;12(3):406. doi: 10.3390/healthcare12030406 (PMC10855396; doi:10.3390/healthcare12030406)
Supplement: Supplementary file 1 [file healthcare-12-00406-s001.zip › healthcare-2821180-supplementary.pdf]

Supplementary Table S1. Breastfeeding tips for women with diabetes.

1. **Breastfeed often**, according to baby's demands.
2. If possible, try to **breastfeed in the delivery room**.
   - Breastfeeding in the delivery room can reduce baby's chance of experiencing neonatal hypoglycemia.
   - If you believe you will not be able to breastfeed in the delivery room, you can, as 36 weeks gestation, manually express milk and store it in the freezer for use afte delivery.
3. Colostrum is the initial milk produced, which is thick and sticky, and is excreted in very small quantities. It transitions into mature milk (waterier and more abundant) 2-3 days after birth. After a caesarean delivery or a diabetic pregnancy, this transition may be delayed up to 4-5 days after delivery.
   - The increase in milk volume can sometimes cause engorgement in the breasts. Breastfeed often to allow the baby to remove as much milk as possible. After breastfeeding, you can place a cold compress on the breast for 15-20 minutes to ease the discomfort, and to prevent further engorgement. This situation should resolve within 2-3 days.
   - **Breast infection (mastitis) tends to worsen quickly in diabetic women, so it important to pay attention to the symptoms and treat them quickly!** If you suspect that you have developed mastitis (a portion of a breast has become especially sensitive, red, and warm to the touch), it is imperative to remove as much milk as possible from the infected breast, especially from that sensitive are – to release the blockage that has formed. Warm the breast before pumping or breastfeeding to make it easier to empty it, and rest as much as possible.
     If the situation does not improve within 24 hours, or if you develop a fever and flu like symptoms, consult your doctor, who may recommend antibiotics for the infection.
     - If your doctor has prescribed antibiotics, probiotics can be taken two hou after the antibiotic dose, to reduce the chance of developing thrush on th breast or in the baby's mouth.
4. Keeping your baby in the room with you at night will allow you to recognize and respond to baby's early-stage hunger cues before baby becomes frustrated and cries. Additiona frequent breastfeeding will encourage your body to produce more milk.
5. Spend as much time as possible "**skin-to-skin**" with your baby - meaning that the skin mom's chest and baby's chest are touching. This promotes frequent breastfeeding, calr baby (and mom), and helps to regulate both baby's body temperature and breath rate.
6. **Latching baby to the breast:**
   position baby's nose in front of your nipple, and keep baby's tummy close to your body (baby's spine should stay aligned from head to pelvis). Once baby has latched onto the breast, check that baby's chin is sunk into your breast tissue.

7. Make sure your body is supported (back, arms, legs, hands…) so that you will be able t remain pain free, by staying in exactly the right position for the whole feed.

8. Signs of proper breastfeeding:

   - Babies tend to feed 8-12 times per 24 hours.
   - The average time for a full feed (not including burping or diaper change) can ran from 7 to 45 minutes.
   - **Breastfeeding should not hurt.** If you are experiencing pain past the first 10 seconds after the initial latch, the latch is probably poor, and is therefore both hurting you, and not efficiently supplying baby with milk. Ask for help and instruction!
   - Check baby is producing enough wet and soiled diapers per 24 hours:
     - Day 1: minimum of 1 soiled diaper (black poop, looks a little like tar) + 1 wet diaper.
     - Day 2: minimum of 2 soiled diapers (black poop, looks a little like tar) + [illegible] wet diapers.
     - Day 3: minimum of 2 soiled diapers (greenish colored poop) + 3 wet diapers.
     - Day 4: minimum of 2 soiled diapers (greenish colored poop) + 4 wet diapers.
     - Day 5 through 30: minimum of 3 soiled diapers (soft, mustard colored poop) + 5-6 wet diapers.
   - If you suspect you are not producing enough milk for your baby, despite frequent feeding and proper latch, ask your doctor to check your thyroid function. Thyroid disfunction is common in women with diabetes, and can make it difficult for the body to produce enough milk.

9. Exclusive breastfeeding burns 500-1000 calories per day!

   - Just like any other exercise that burns calories, it can also cause low blood suga Low blood sugar around breastfeeds is especially common during the first two months after birth. Make sure you always have sugar within reach!

10. To take care of baby, **mom must be taken care of too**! Pay attention to your emotiona wellbeing and seek professional help if the need arises.

   - **Taking proper care of yourself and your diabetes is crucial even after the pregnancy is over. This is not always an easy task to bear.** Pay special attention to a <u>poor emotional state, which does not resolve within a month after birth</u>. <u>Ask your partner to pay attention as well,</u> to significant emotional or behavioral changes in you, so they can help you get help if you need it.

…One more thing; if your you've got a question, ask!

If you are unsure, if you’re in pain, if you're worried – ASK FOR HELP
from the hospital staff or hire a private counselor to meet you at home or in the hospital.

Supplementary Table S2. Association analysis for any breastfeeding at six-months post-partum.

Characteristics	Any breastfeeding until six-months post-partum (n=26)	Any breastfeeding beyond six-months post-partum (n=26)	P-value*
Type 1 Diabetes, n (%)	16 (62)	16 (62)	1.000
Type 2 Diabetes, n (%)	10 (38)	10 (38)	1.000
Maternal age (years), mean±SD	33±5	33±5	0.594
High School graduate, n (%)	14 (54)	16 (62)	0.779
Nulliparous, n (%)	8 (31)	3 (12)	0.173
Smoker, n (%)	4 (15)	2 (8)	0.668
Cesarean delivery, n (%)	16 (62)	7 (27)	0.030
Birthweight (grams), mean±SD	3452±411	3399±63	0.689
Gestational age at delivery, median (min-max)	38 (37-39)	38 (34-39.2)	0.080
Newborn jaundice, n (%)	3 (12)	6 (23)	0.465
Newborn hypoglycemia, n (%)	6 (23)	5 (19)	1.000
Breastfeeding previous experience, n (%)	9 (35)	23 (88)	<0.001
Breastfeeding in the delivery room, n (%)	4 (15)	19 (73)	<0.001

*P-value is significant at <0.05.

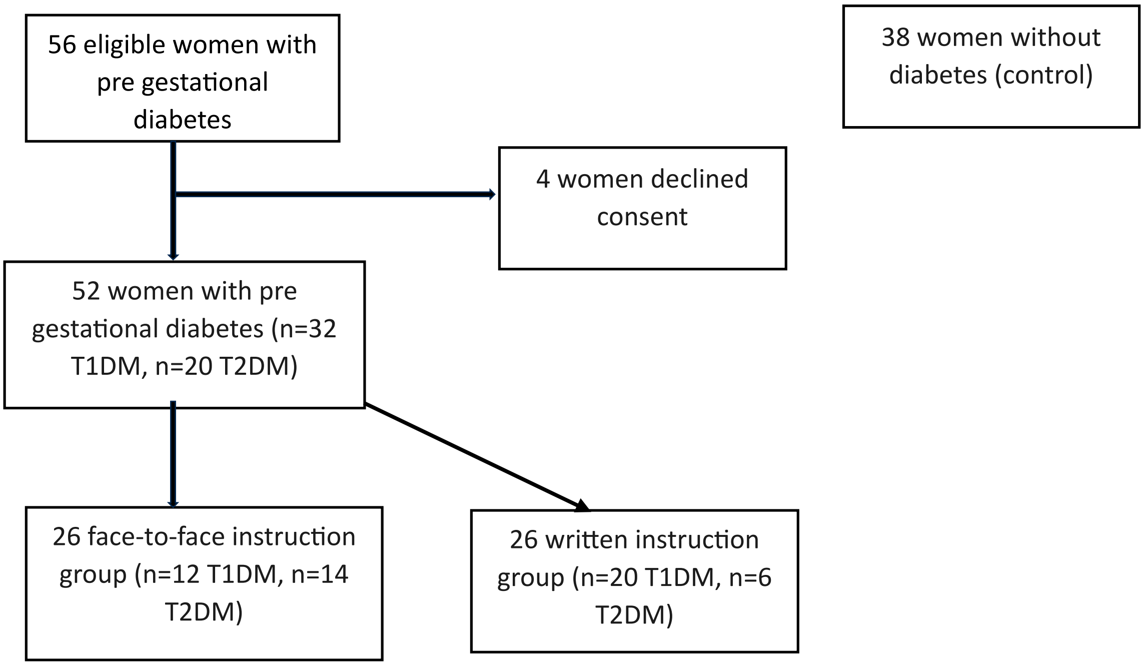

T1DM: type 1 diabetes mellitus; T2DM: type 2 diabetes mellitus.

Supplementary Figure S1. Flow chart showing the recruitment of the subjects.
